# Estimation of Fluoride Release from Various Dental Materials in Different Media—An *In Vitro* Study

**DOI:** 10.5005/jp-journals-10005-1033

**Published:** 2009-04-26

**Authors:** Anant G Nigam, JN Jaiswal, RC Murthy, RK Pandey

**Affiliations:** 1 Ex-PG Student, Department of Pedodontics, KGMC, Lucknow, Uttar Pradesh, India; 2 Ex-Professor and Head, Department of Pedodontics, KGMC, Lucknow, Uttar Pradesh, India; 3 Scientist and Head, Metal Analysis Laboratory, ITRC, Lucknow, Uttar Pradesh, India; 4 Professor and Head, Department of Pedodontics, KGMC, Lucknow, Uttar Pradesh, India

**Keywords:** Fluoride, glass ionomer, compomer, composit resin.

## Abstract

*Purpose:* This study was performed to study the pattern of fluoride release from glass ionomer cement, a resin modified glass ionomer cement, a compomer and a composite resin when stored in different storage media.

*Methods:* A total of 60 samples (Tablets of diameter 8.6 ± 0.1 mm and thickness 1.65 ± 0.1 mm) were prepared (15 samples for each material) pertaining to 4 different materials used. Five samples of each material were suspended in 4 ml of each studied solution. The studied media were deionized water, artificial saliva and solutions for pH-cycling (demineralizing solution pH 4.3 and remineralizing solution pH 7.0). The total experimental period was fifteen days the readings were taken after day 1, day 2, day 5, day 9 and day 15 using ORION fluoride ion specific electrode.

*Results:* Significant variations were found in the amount of fluoride release from all the materials in different storage media.

Glass ionomer cement released significantly higher amounts of fluoride (p < 0.001) in all storage media. The difference between composite resin and other materials was also very significant (p 0.001) where composite resin released very less fluoride in all the media.

*Conclusion:* From this study it was concluded that the greatest amount of fluoride release was from ART glass ionomer cement in all the media followed by resin modified glass ionomer cement, compomer and composite resin in decreasing order. The pattern of fluoride release was similar for all the examined materials.

Maximum amount of fluoride release was observed in pH cycling model for all the materials followed by deionized water and artificial saliva in decreasing order. With this it can be concluded that pH strongly affects fluoride release from dental restorative materials.

## INTRODUCTION

Caries prevention and eradication has been the greatest challenge that the dentists world over have been facing. The prime objective of dental treatment today is not only caries restoration but to make an attempt to induce changes in the dental tissues that may resist the initiation of carious process itself.

The role of fluoride in preventing dental caries has been well-documented. It is a well understood fact that fluorides have an anti-cariogenic property and it prevents initiation and progression of caries by forming a caries resistant complex with inorganic portion of tooth material. So dental restorative materials which contain fluoride in their formulation and are able to provide sustained release of fluoride might prove to be helpful in the inhibition of dental caries in adjacent teeth as well as prevention of secondary caries in the pre-restored teeth as well.

These days various restorative materials containing fluoride in their formulation are available in the market. These materials such as glass ionomer cements, Resin modified glass ionomers, compomers and composites are able to release fluoride ions in the oral environmental. The fluoride ions released combines with hydroxyapatite crystals in the inorganic portion of the tooth to form fluorapatite which is a caries resistant complex.

The rate and pattern of release of fluoride ions from restorative materials depends on various factors such as temperature, pH of the environment, mixing technique, powder liquid ratio, media surrounding the material, area that is exposed to the oral environment, etc.

Most of the studies have been performed to study the pattern of fluoride release from various restorative materials in neutral pH or inert solutions like deionized or double distilled water. Very few studies have been conducted on fluoride release pattern during the caries experience which actually occurs in the mouth.

The present investigation was undertaken to study the effect of change in pH on the pattern of release of fluoride ions from a glass ionomer cement, resin modified glass ionomer cement, a compomer and a composite which was compared with fluoride release pattern of these materials in deionized double distilled water and artificial saliva at constant temperature.

It is felt that the result from present investigation will enable a pedodontist and the dental professional to know and critically analyze the materials on the basis of their fluoride releasing property.

## MATERIAL AND METHOD

**Fig. 1.**
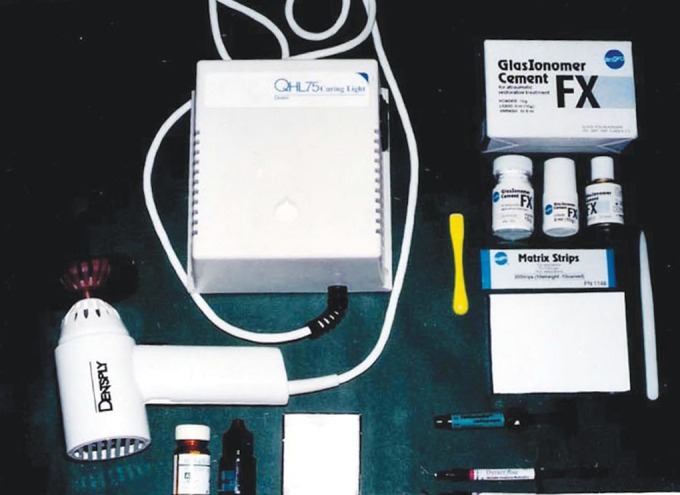
Dental material used

The study comprised of a total of sixty samples divided in four groups pertaining to four different dental materials used. The dental materials used were-glass ionomer cement, a resin modified glass ionomer cement, a compomer and a fluoride releasing composite (Fig. 1). These materials were procured directly from the market. Each material was having a batch number and date of expiry printed on it. The materials tested are given in Table 1.

**Fig. 2.**
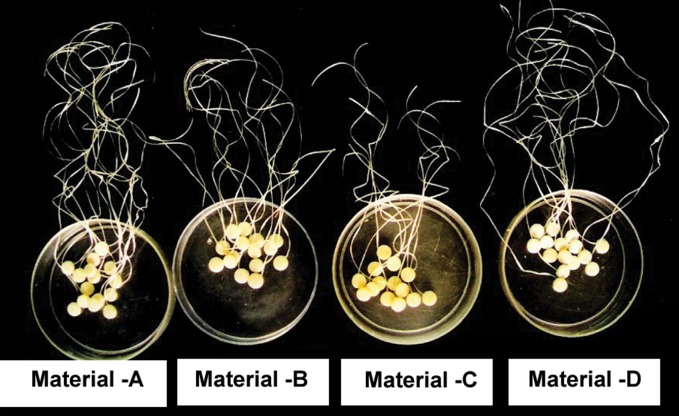
Different samples

**Fig. 3.**
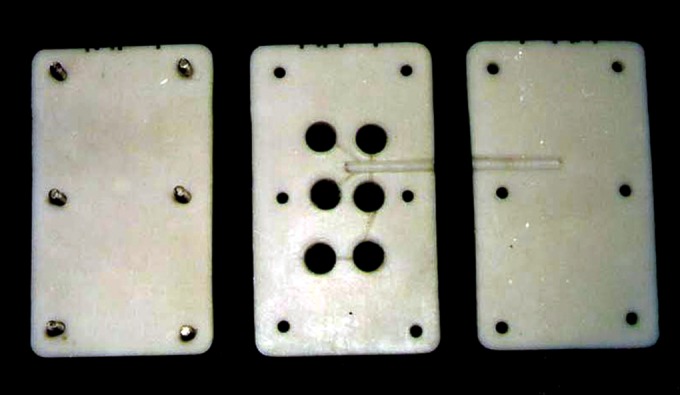
Teflon mould

Sixty samples, 15 for each material (5 for each medium) were prepared as thin disks of diameter 8.6 mm and thickness 1.65 mm (Fig. 2) at room temperature according to ISO specification # 7489 using teflon moulds (Fig. 3). The materials were manipulated according to the manufacturer’s recommendations, placed in telfon moulds and pressed between two telfon plates. Paraffin dental floss (Oral-B waxed) was incorporated into the samples during setting to suspend the samples in the respective medium. The material I was set by chemical reaction whereas materials II, III and IV were light cured (Dentsply, Germany) for recommended time periods.

**Table 1:** Table 1: Materials used in the study

*Group*		*Restorative*		*Material type*		*Manufacturer*		*Batch no.*		*Shade*		*Date of*		*P/L*		*Type of*
		*material*										*expiry*		*ratio*		*curing*
I		Shofu-FX		Glass ionomer		Shofu-Inc.		0799		Universal		June, 2003		2.8:1		Self cure
				cement		Japan										

II		Fuji-II-LC		Resin modified		GC Corp.,		6071		A3		June, 2002		3.2:1		Light cure
				glass ionomer		Japan										
				cement												

III		Dyract (flow)		Polyacid modified		Dentsply		6071		A3		March, 2001		Syringe		Light cure
				composite resin		Detrey,										
				(compomer)		Germany		911								

IV		Heliomolar		Composite		Vivadent		B 40700		A2		Oct., 2004		Syringe		Light cure
		(Radiopaque)				Liechtenstein,										
						Germany										

These samples (fifteen in number for each group) were randomly divided into 3 subgroups consisting of 5 samples each.

The samples of each subgroup were placed in a predetermined storage media as described below:

Subgroups    Ia, IIa, IIIa, IVa    Medium A

Subgroups    Ib, IIb, IIIb, IVb    Medium B

Subgroups    Ic, IIc, IIIc, IVc    Medium C

Medium A was deionized water.

Medium B was artificial saliva.

**Fig. 4.**
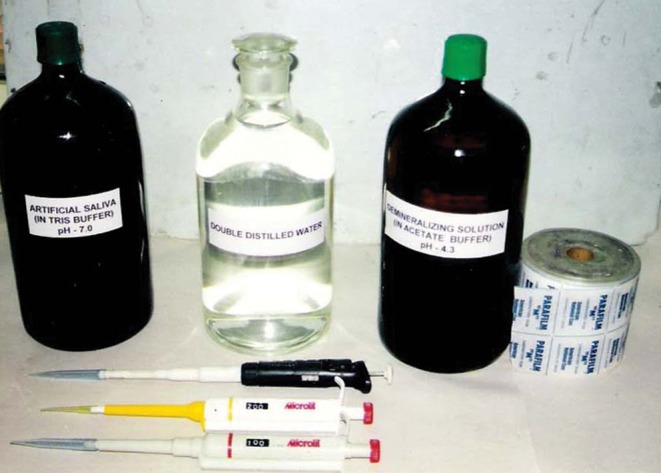
Storage medium

Medium C was pH cycling model that consisted of alternating demineralizing (C1) and remineralizing (C2) solutions at the intervals of 6 hours and 18 hours respectively. The composition of storage media are given in Table 2 (Fig. 4).

Each sample of subgroups Ia, IIa, IIIa, IVa and Ib, IIb, IIIb, and IVb were then placed in individual polypropylene vials (Tarsons Inc 18 × 100 mm) containing 4 ml of their respective medium. The vials were then covered with Parafilm "M" laboratory film and were placed in incubator (Scientronics, India) at a constant temperature of 37 ± 0.5°C for twenty-four hours.

At the end of 24 hours the specimens were taken out of the vials by pulling out the string attached to them. The specimen were washed in 1 ml of flowing distilled water which was added to previous 4 ml of storage media to make it to 5 ml. The specimens were then again placed in 4 ml of fresh storage media and placed in the incubator till the next reading was taken after predetermined time interval subsequent transfers were performed for all the samples in the same manner at the end of 2, 5, 9 and 15 days.

**Flow Chart 1:**
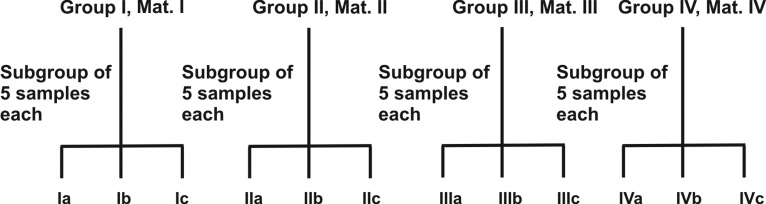
Subgrouping of samples

**Table 2:** Table 2: Composition of different storage media

**Medium A** was deionized water. It was procured from Milli-Q, Millipore system in the laboratory		
Medium B artificial saliva		
Ca 1.5 mM		(CaCl_2_ 0.1665 g/l)
PO_4_ 0.9 mM		(NaH_2_PO_4_ 0.133 g/l)
KCl 150 mM		(KCl 11.184 g/l)
Tris buffer 20 mM		(2.4228 g/l)
NaN_3_ 0.02%		
pH was adjusted to 7.0 by addition of dilute HCl and the total solution was made to one liter.		
Medium C1 demineralizing solution		
Ca 2.0 mM		(CaCl_2_ 0.22 g/l)
PO_4_ 2.0 mM		(NaH_2_PO_4_ 0.2399 g/l)
Acetate buffer 75 mM		(NaCH_3_COO 6.152 g/l)
NaN_3_ 0.02%		
pH was adjusted to 4.3 by the addition of dilute HCl and dilute NaOH and the total solution was made to one liter.		
The composition of remineralizing solution medium C2 was same as artificial saliva.		

Each sample of subgroups Ic, IIc, IIIc and IVc were placed in demineralizing solution (C1) for 6 hours and same sample was transferred to remineralizing solution (C2) for 18 hours for pH cycling. At the end of day one the two solutions were mixed and sample was transferred to the fresh storage medium for next reading. The subsequent readings were taken at the end of day 2, 5, 9 and 15. However, the transfer of the sample to demineralizing and remineralizing solution were done at 6 hourly and 18 hourly interval respectively.

**Fig. 5.**
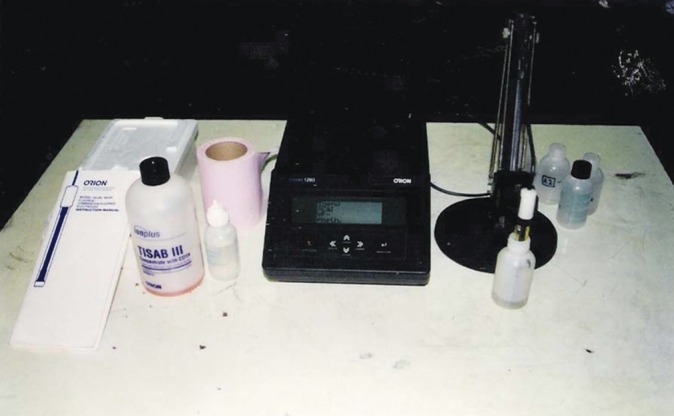
ORION digital ion analyzer

Estimation of fluoride leached in various solutions was done by using ORION digital ion analyzer, model (1260), equipped with combination ORION fluoride ion specific electrode (96-09) (Fig. 5). After calibrating the electrode with standard fluoride solution of 1 and 10 ppm, estimation of fluoride release in each samples solution was done by taking 5 ml of sample aliquot to which 0.5 ml of TISAB-III solution was added. This was done to eliminate any interference from other ions such as Al^3+^, Na^+^, Sr^+^, etc. This solution was stirred for 60 seconds and then the tip of the calibrated electrode was completely dipped in the solution. When stable reading was displayed in ppm on the digital screen, it was noted down. Fluoride release was calculated by using the formula.

(Fluoride release = Fµg/ml × volume of solution/Area of sample in cm^2^)

Surface area (cm^2^) = 2 Πr (r + h)

During the estimation the temperature of the laboratory varied form 20°C to 24°C.

## RESULT

**Fig. 6.**
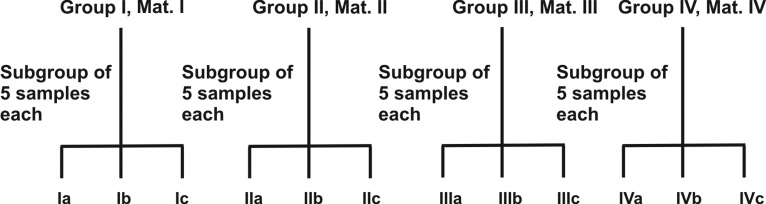
Mean fluoride release (µgF/cm^2^) by various dental restorative materials in deionized water at different time intervals

**Fig. 7.**
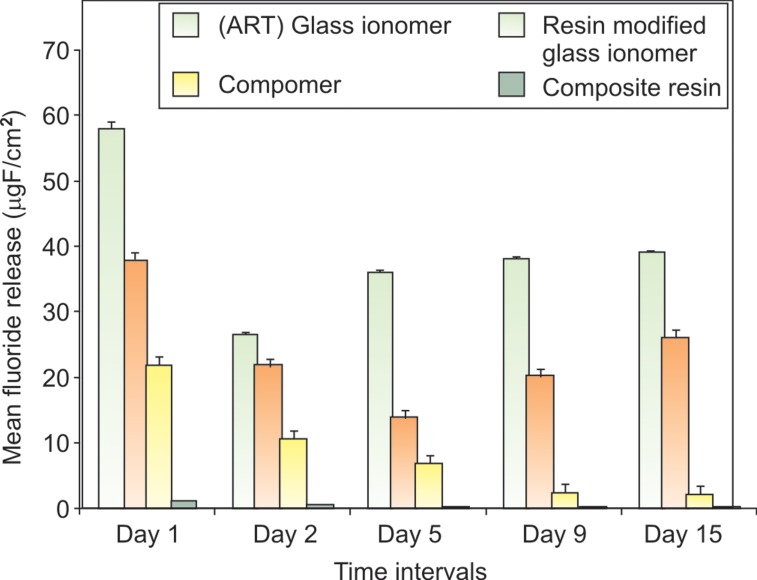
Mean fluoride release (µgF/cm^2^)by various dental restorative materials in artificial saliva at different time intervals intervals

**Fig. 8.**
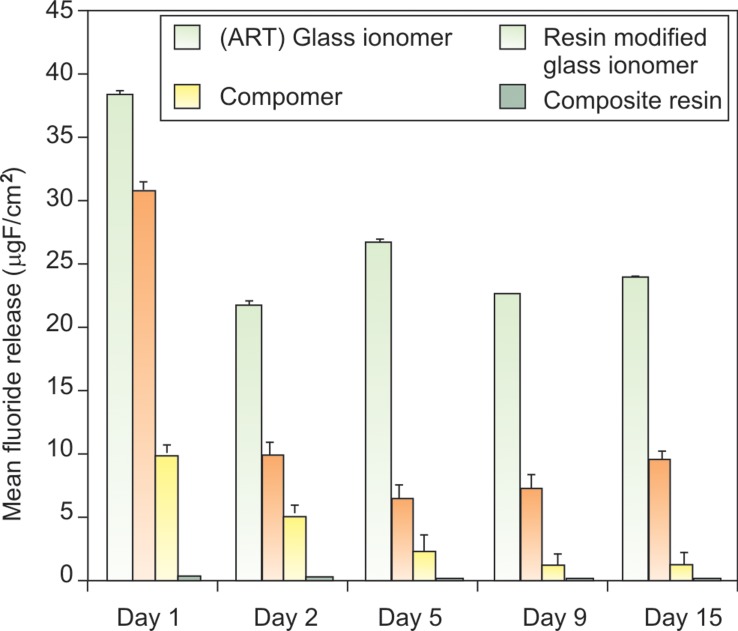
Mean fluoride release (µgF/cm^2^)by various dental restorative materials in pH cycling model at different time intervals

The fluoride release values of various materials studied in different media are given in Table 3 (Figs 6 to 8). Although great differences in the amounts of fluoride released from the materials exist, the pattern was similar in various media.

The most important observations were: a significant variation in the amount of fluoride released from all the materials in water *vs* pH cycling model (p < 0.001) and in artificial saliva *vs* pH cycling model (p < 0.001), except in the case of composite, where the fluoride release was not significantly different in water vs pH cycling model at the end of day one.

Among the examined materials ART glass ionomer cement released significantly higher amounts of fluoride than various other dental restorative materials in all storage media. The difference between composite and the other dental materials was also very significant (p < 0.001). Composite released significantly less fluoride than other materials at all time intervals. Significant difference (p < 0.001) was observed in the amounts of fluoride release from ART glass ionomer cement vs resin modified glass ionomer cement in all the storage media. The difference in fluoride released from compomer in deionized water vs artificial saliva and in pH cycling model vs deionized water and artificial saliva were highly significant (p < 0.001).

**Table 3:** TABLE 3: Mean fluoride release from different materials (µg F/cm^2^) in different media

*Days*		*1 *		*2 *		*5 *		*9 *		*15 *
Glass ionomer cement (Material I)										
Medium A		57.97 + 1.22		26.622 ± 0.731		36.034 ± 0.820		38.196 ± 0.900		39.156 (2.63)
Medium B		38.51 ± 1.354		21.92 ± 0.664		26.768 ± 0.880		22.644 ± 0.897		23.96 ± 0.723
Medium C		82.158 ± 1.766		54.308 ± 1.622		63.45 ± 0.483		69.818 ± 1.975		74.472 ± 0.830
Resin modified glass ionomer cement (Material II)										
Medium A		37.85 ± 1.205		22.018 ± 1.207		14.054 ± 1.070		20.398 ± 1.297		26.198 ± 1.333
Medium B		30.884 ± 1.045		10.064 ± 0.717		6.618 ± 0.785		7.464 ± 0.657		9.564 ± 1.201
Medium C		63.346 ± 0.758		39.352 ± 1.480		51.308 ± 0.901		54.996 ± 0.533		55.43 ± 0.336
Compomer (Material III)										
Medium A		21.938 ± 1.069		10.65 ± 2.178		6.894 ± 1.056		2.456 ± 0.381		2.214 ± 0.432
Medium B		9.99 ± 1.129		5.378 ± 0.988		2.464 ± 0.539		1.258 ± 0.225		1.054 ± 0.169
Medium C		39.226 ± 1.086		29.09 ± 0.481		33.188 ± 0.661		30.434 ± 0.323		39.552 ± 1.098
Composite resin (Material IV)										
Medium A		1.022 ± 0.149		0.222 ± 0.024		0.168 ± 0.028		0.114 ± 0.026		0.06 ± 0.013
Medium B		0.468 ± 0.074		0.066 ± 0.017		0.06 ± 0.009		0.078 ± 0.015		0.06 ± 0.013
Medium C		1.22 ± 0.101		1.118 ± 0.124		1.036 ± 0.148		0.862 ± 0.127		0.848 ± 0.074

## DISCUSSION

Fluoride release from restorative materials is a complex process involving several phases, such as diffusion of water into the material, dissolution of fluoride in the solid and diffusion of fluoride ions out of the material into the solution.

The rate of fluoride release from a material can be affected by several factors such as the media in which the samples are stored, temperature, area that is in contact with storage medium and powder liquid ratio of the material.^1^ In the present study, the temperature was kept equal to the normal body temperature by placing the samples in an incubator at 37°C. In addition, the samples were made to suspend in the storage medium with the help of dental floss attached to them so that they do not cling to the walls of the polypropylene vials,^2^ thus, allowing uniform wetting of the sample by the storage medium. The samples were prepared strictly according to manufacturers recommendations by a single operator to rule out any individual error. The basic aim of the study was to assess the amount and pattern of fluoride release from various esthetic dental materials when stored in different media.

Deionized water was chosen for the experiment as it provided the baseline of fluoride release potential in unstimulated conditions. This is in agreement with the earlier studies.^1^,^3^-^7^

Artificial saliva was chosen as a second medium for fluoride leaching so as to simulate to an extent the natural oral environmental conditions, although, duplicating exactly the properties of human saliva is impossible due to the inconsistent and unstable nature of natural saliva. So the development of artificial saliva is essential for welljustified and controlled experiments.^21^

The third medium was pH cycling model^8^ that consisted of alternation demineralizing (pH-4.3) and remineralizing (pH-7.0) solutions which represent a dynamic situation that is commonly encountered in the mouth. The pH cycling that occurs in dental plaque affects the release of fluoride ions from the restorations found in vicinity of dental plaque and can have a great influence on the fluoride release pattern of the restorations. So, *in vitro* evaluation of dental materials that release fluoride should take into account the pH cycling in dental plaque.

In the present study common finding was that the highest fluoride leaching occurred during the first 24 hours from all materials.^9^-^12^ The dental materials continued to release fluoride until the end of the experiment. This finding gains support from the earlier reports.^1^-^3^,^6^,^13^,^14^

The elution of fluoride occurs as two different processes. The first process is characterized by an initial burst of fluoride release from surface, after which the elution markedly reduced. This early release is further confirmed by recent investigations that the average hourly release rates during the first day were higher during the first hour.^15^ The first process is accompanied by a second bulk diffusion process in which small amounts of fluoride continued to be released into the surrounding media for period’s up to atleast two to two and a half years.^9^,^10^

In the present study the values of fluoride release in deionized water and in artificial saliva were consistently different which is in agreement with the earlier studied.^4^,^15^-^17^,^19^ The higher values were observed in deionized water. The lower values observed in artificial saliva may be due to the presence of cations and anions in artificial saliva which may have an ionic effect on the solubility of the material.

Fluoride release was found to be consistently higher in pH cycling model as compared to water and artificial saliva as reported earlier.^19^-^21^ These observations are in agreement with previous investigations who observed that the acidic conditions during the daily demineralization period probably increase the release of fluoride through chemical erosion. This explains that low pH resulted in greater release of fluoride.

The results were statistically significant for all dental materials. The significant difference in the amounts of fluoride released in pH cycling vs saliva and water could be attributed to the fact that the dissolution of the dental materials was dependent on the solvent.

The resin modified glass ionomer cement released less fluoride than (ART) glass ionomer cement. The resin network could also reduce the diffusion of water into cement, thus, reducing the elution of unbound fluoride in the material matrix.

The release of fluoride by compomer was slightly more than composite but significantly less than Resin modified glass ionomer cement and ART glass ionomer cement.

The least amount of fluoride was released from composite resin. This is in agreement with the previous reports.^3^,^6^ It seems that fluoride compound added to the composition of composite resin lead to low fluoride release. Even the use of low pH solution did not produce the significant release of fluoride from composite.

It is evident from the study that with an increase in the amount of resin component and decrease in polyacid and glass filler content of the material, a decrease in fluoride release values is seen. The pH cycling model is also unable to dissolve the resinous component of the material to an extent that it can release substantial fluoride.

## CONCLUSION

The present study was conducted with a view to evaluate and compare fluoride releasing property of glass ionomer cement, resin modified glass ionomer cement, polyacid modified composite resin and composite resin. The study led to the following conclusion.

1. All the materials released fluoride ions during the entire experimental period. Maximum fluoride release was observed on day 1 for all the materials in all the studied media.
2. Atraumatic restorative treatment (ART) technique glass ionomer cement released maximum amount of fluoride ions at different time intervals in the different storage media. This was followed by resin modified glass ionomer cement, polyacid modified composite resin and composite resin in decreasing order.
3. Maximum fluoride ion released was observed in pH cycling model for all the studied restorative materials. This was followed by deionized water and artificial saliva in decreasing order.
